# Pediatric Exposures to Ionizing Radiation: Carcinogenic Considerations

**DOI:** 10.3390/ijerph13111057

**Published:** 2016-10-28

**Authors:** Kristy R. Kutanzi, Annie Lumen, Igor Koturbash, Isabelle R. Miousse

**Affiliations:** 1 Department of Environmental and Occupational Health, College of Public Health, University of Arkansas for Medical Sciences, Little Rock, AR 72205, USA; ikoturbash@uams.edu; 2 Division of Biochemical Toxicology, National Center for Toxicological Research, Jefferson, AR 72079, USA; Annie.Lumen@fda.hhs.gov

**Keywords:** radiation, children’s health, cancer, computed tomography

## Abstract

Children are at a greater risk than adults of developing cancer after being exposed to ionizing radiation. Because of their developing bodies and long life expectancy post-exposure, children require specific attention in the aftermath of nuclear accidents and when radiation is used for diagnosis or treatment purposes. In this review, we discuss the carcinogenic potential of pediatric exposures to ionizing radiation from accidental, diagnostic, and therapeutic modalities. Particular emphasis is given to leukemia and thyroid cancers as consequences of accidental exposures. We further discuss the evidence of cancers that arise as a result of radiotherapy and conclude the review with a summary on the available literature on the links between computer tomography (CT) and carcinogenesis. Appropriate actions taken to mitigate or minimize the negative health effects of pediatric exposures to ionizing radiation and future considerations are discussed.

## 1. Introduction

Exposures from the largest nuclear disasters in human history—first in Hiroshima and soon after in Nagasaki—provided clear evidence that ionizing radiation (IR) is a human carcinogen. Significant increases in blood, breast, and other cancers have been observed in A-bomb survivors [1]. The results of these epidemiological studies have also demonstrated that exposure to IR during childhood may result in an increased excess risk of cancer compared to adults, pointing towards higher sensitivity to radiation-induced cancers in children. Among exposed children, the incidence of leukemia rose dramatically just a few years after exposure [2]. Studies from Chornobyl (Ukrainian transliteration) further expanded our knowledge on increased sensitivity of children to IR, as indicated by the substantial increase in the relative risk of thyroid cancer in children and adults who were exposed to high thyroid doses (over 1 Gy) in their childhood [3].

It is now generally accepted that children are more sensitive to radiation than adults, specifically with higher relative risk of cancers including leukemia, brain, breast, skin, and thyroid cancers following exposures [4]. In part, this is because of the radiosensitivity of their developing organs and tissues [5,6]. Also, the longer post-exposure life expectancy increases the lifetime risks of developing radiation-induced malignancies [7,8]. This is becoming important because 70% to 80% of all children diagnosed with cancer have long-term survival [9]. In this review, we will summarize the current knowledge on the risks of radiation-induced cancers as a consequence of pediatric exposures.

## 2. Evidence from Accidental Exposures

The first indications that children were at increased risk compared to adults of developing malignancies from radiation came from studying populations exposed to radioactive fallout from nuclear weapons and power plants.

### 2.1. Leukemia

Leukemia was the first radiation-induced cancer reported in A-bomb survivors and was prevalent predominantly in children. The first evidence of pediatric leukemia was reported 3 years after exposure and reached its peak during the 6–8 year period [10,11]. Analysis in the Life Span Study of Japanese atomic bomb survivors indicated 310 deaths due to leukemia during the period of 1950–2000 in 866,111 people. In this study, the excess relative risk (ERR, the proportional increase of risk) for leukemia in children under age 10 at the time of exposure peaked at about 70 per Gy of irradiation, while the ERR among those who were exposed at the age of 30 and older was around 2 [11].

A number of studies have investigated the association between exposures to natural sources of radiation and childhood leukemia. Some of those studies identified positive associations [12,13], although some problems with data interpretation were raised [14]. A recent comprehensive record-based case-control study performed by Kendall and colleagues demonstrated a statistically significant leukemia risk associated with exposure to natural sources of gamma radiation. Specifically, in the cohort of 27,447 cases of childhood leukemia and 36,793 cancer-free controls, the authors reported 12% ERR of childhood leukemia per millisievert of cumulative red bone marrow dose of radiation [15].

### 2.2. Thyroid Cancer

Ionizing radiation has been linked to thyroid cancer, with some studies reporting that ~9% of diagnosed thyroid cancers are due to radiation exposure [16]. The developing thyroid gland is particularly susceptible to ionizing radiation. Thyroid cancer was the most common type of solid tumors to develop among A-bomb survivors who were under 20 years of age at the time of the event [17]. In their study, Thompson and colleagues listed all cases of tumors for the 1958 to 1987 period and found that thyroid cancer was a particularly common finding in children who were under 10 in 1945, with the effect decreasing with age. A number of epidemiological studies also demonstrated increases in thyroid cancers in children and young adults who were exposed to high thyroid doses (>1 Gy) in childhood as a result of ^131^I released during the Chornobyl accident [3,18,19]. The first cases of thyroid cancer due to irradiation from the Chornobyl explosion were observed as early as five years after the accident [20]. Importantly, the highest rates of thyroid cancer were observed among children who were 5 years of age or less at the time of exposure, suggesting vulnerability of the thyroid gland in this age range. The risks decline with age, and it is generally accepted that the risk of developing thyroid cancer due to radiation are diminished when exposure occurs after age 15 [21]. Furthermore, elevated levels of thyroid cancer were not observed in children who were born after the accident, clearly indicating that thyroid cancer risks were primarily associated with the release of ^131^I, which has a short half-life of 8 days [22]. Besides thyroid carcinomas, significant increases in benign thyroid follicular adenomas were also observed in young adults [23].

The recent accident at the nuclear power plant in Fukushima, Japan, has raised a number of concerns regarding potential carcinogenic effects on the thyroid gland as a result of the radioactive iodine isotopes that were released. A report issued in 2013 by the United Nations Scientific Committee on the Effects of Atomic Radiation in response to this accident concluded that individuals exposed as adults were not expected to develop thyroid cancer above the background level, while some increase was to be expected for infants and children [4]. A recent study from Tsuda et al. did indeed find an increase in thyroid cancer among children and adolescents four years after the event [24]. However, doubts have been raised about the biasing effect of increased screening on the rates observed [25]; a sudden increase in screening might have detected other unrelated diseases that would have otherwise remained undiscovered, artificially inflating numbers [26,27].

### 2.3. Other Cancers

In addition to leukemia and thyroid cancer, an overall increase in the incidence of solid tumors was observed in A-bomb survivors [17]. Increased breast cancer rates and breast cancer-related mortality rates were reported in female A-bomb survivors who were children at the time of exposure [17,28]. There are also indications that the rates of breast cancer in Belarus and Ukraine increased after the 1986 Chornobyl incident, especially in women who were younger at the time [29]. Other types of cancers encountered in those who were under 20 years of age during the events at Hiroshima and Nagasaki were non-melanoma skin cancer, cancer of the urinary organs and kidney, and ovarian cancer in females [17]. There is also evidence that exposure to low doses of radiation can have negative impacts on hepatocytes. Data from epidemiological studies on A-bomb survivors show significant increases in liver cancer rates and the standardized mortality ratio (SMR) in women exposed to low doses of radiation (colon radiation doses below 0.1 Gy) and in men exposed to low and high doses of radiation (colon radiation doses below 0.1 Gy and doses in the range of 0.1–4 Gy, respectively) [28].

### 2.4. Actions Taken and Future Considerations

In 1982, the United States Food and Drug Administration (FDA) approved and recommended the use of potassium iodide (KI) as a protective agent against radioiodine exposure to mitigate the risk of thyroid cancer. The mode of action of KI is to block the thyroidal uptake of radioiodines, hence diluting its distribution and biological incorporation. Based on the aftermath data on radioiodine exposure and its associated thyroid cancer risk following the 1986 Chornobyl reactor accident, the FDA further updated its recommendations and issued revised guidelines in 2001 [30]. The recommended treatment doses of KI are 16 mg/day for newborns from birth to 1 month, 32 mg/day for infants over 1 month after birth through 3 years of age, and 65 mg/day for adults. As summarized in the guidance, 0.05 Gy was recommended as the lowest radiation exposure intervention threshold for KI administration. For optimal exposure prevention, the prophylaxis dose—the same as the treatment dose—was recommended to be administered daily until the threat of radioiodine exposure subsided. It is advised that KI be administered before or immediately after an exposure event. The protective effect of KI increases considerably if taken within 3 or 4 h of exposure and lasts for 24 h. However, 12 out of 3214 neonates in Poland treated with KI for radioiodine exposure after the Chornobyl accident developed transient hypothyroidism. Therefore, the current guidance suggests a single dose of 16 mg/day of KI for neonates and has advised that repeat dosing be avoided to reduce the risk of iodine-induced hypothyroidism. Thyroid function of neonates and infants treated with KI, especially within the first month of life, is recommended to be monitored and treated, should hypothyroidism ensue.

A review of the functional aspects of the thyroid axis across life-stages provides supporting evidence that thyroid functions in infants and children are accelerated compared to adults [31]. The current recommendations for KI dose and duration were adjusted downward by age group in consideration of body size, assuming thyroid function scales linearly with development and age. Future considerations could include an initiative to evaluate the suitability of accounting for thyroid function differences with age and body-size changes in order to derive appropriate KI dosing regimens for the youth. Due to ethical considerations, it is difficult to conduct controlled clinical studies in infants and children to estimate optimal KI dosing normalized for thyroid function differences. However, advances in the field of computational toxicology allow for the development of tools that can extrapolate findings from controlled animal studies to humans. Such an approach can be utilized to determine safe and effective KI dosing in the young.

## 3. Exposure from Treatment Modalities

Children can also be exposed to radiation when they develop disorders for which the treatment regimen requires radiation therapy (RT). Although the risk of second primary malignancies (SPMs) is elevated for all childhood survivors, the risk is further heightened for those who received RT [32,33]. RT has been used for over 100 years and while short-term adverse effects were rapidly described, the study of adverse long-term effects is still an active field of investigation.

### 3.1. Cancer Risks Associated with RT

One of the first studies on the long-term outcomes of RT was published in 1950 by Duffy and Fitzgerald [34]. In the early 20th century, irradiation was a common treatment for enlarged tonsils. The two physicians analyzed patients with thyroid cancer with onset under 18 years of age and noted that out of 28 pediatric patients, 10 had received RT for an enlarged thymus between the ages of 4 and 16 months. This led to additional investigations in different cohorts. More researchers identified radiation-related thyroid tumors among adults who had received RT as children for enlarged tonsils, and respiratory or skin infections [35,36,37,38]. Increased incidence of acute leukemia [35] and brain cancers [37] were also identified. However, the number of children having been exposed to radiation for benign conditions is small compared to that receiving RT for life-threatening cancers.

In 2010, there was an estimated 380,000 pediatric cancer survivors in the US [39]. This number is expected to increase as survival rates have been steadily improving over the last decades. Data suggests that some of them will go on to develop a SPM as a result of their treatment. A number of epidemiological studies reported a significant increase in the incidence of treatment-related leukemias, especially acute myeloid leukemia, as a result of RT in childhood [33,40,41]. Also, it should be emphasized that survivors of childhood cancer treated with RT have developed multiple long-term chronic medical conditions that further decreases their quality of life [42].

The location of SPMs is typically a function of the area of the body that was irradiated. For example, SPMs most commonly associated with irradiation of the neck and chest necessary to treat Hodgkin’s lymphoma (HL) are leukemia, non-Hodgkin lymphoma (NHL), thyroid cancer, and breast cancer in women [32,43,44,45]. In fact, under some treatment regimens (≥40 Gy to the chest, without alkylating agents), nearly 30% of women treated for HL as children developed breast cancer by age 55 [44]. Sarcomas can also appear near the original irradiated tumor [46]. The Childhood Cancer Survivor Study (CCSS) has been compiling data from 22,343 childhood cancer survivors over the last 20 years at St Jude Children’s Research Hospital [47]. Of these, 57.3% received RT, with 9.3% having received a maximum dose of at least 50 Gy to the brain and 11.2% having received at least 30 Gy to the chest (December 2015 update). Excess relative risk per Gy of radiation has been calculated for SPMs in the brain, breast, thyroid gland, bone, skin, and salivary gland [48,49,50,51,52,53,54]. The results have been reviewed by Inskip and colleagues [55]. In line with what is known from A-bomb survivors and children treated for benign conditions, the thyroid gland showed the highest excess relative risk at 1.38 per Gy. This was followed by bone (1.32) and skin (1.09). Two separate analyses on that cohort have also linked RT to the development of CNS tumors, mainly gliomas and meningiomas [49,56]. In both studies, the latency period for the development of secondary gliomas was shorter (9 years post radiation) and longer for meningioma (17–19 years after radiation). The rates of CNS tumors are particularly high among children who had leukemia as a first cancer. In addition to girls treated for HL, breast cancer rates were elevated in those receiving RT to the chest for NHL and Wilms tumor. Girls irradiated for bone and soft-tissue sarcoma, whether or not it involved RT to the chest, were also found to be at increased risk [57].

The risk factors making children more sensitive to radiation from therapeutic sources are similar to the ones from accidental exposure to radiation. Additionally, it is estimated that over 8% of pediatric cancers are caused by genetic susceptibilities [58]. These genetic variations that led to the original cancer also further heighten patient susceptibility to developing cancer as a result of RT. For example, most cases of retinoblastoma are caused by a mutation in the gene *RB1* and these children are at increased risk of developing secondary orbital sarcomas as a result of RT [59].

### 3.2. Actions Taken

Despite the heightened risk for developing SPMs, RT for pediatric cancer is a life-saving procedure. While doses of 34–40 Gy on relatively large fields of radiation were historically used, they were reduced to 21–25 Gy by the mid-1980s. Novel approaches are also being implemented to increase effectiveness while reducing side-effects. In an excellent review, Newhauser and Durante discuss the technical advances in pediatric RT in children [60]. Image-guided RT (IGRT) allowed tracking daily changes in tumor size and organ motions. Three-dimensional conformal RT (3D-CRT) is an improvement over traditional X-ray photon radiation, because 3D-CRT entails precisely calculating the size and shape of the tumor in order to deliver the radiation dose in the most precise way possible. Intensity modulated RT (IMRT) takes advantage of the information gained about the shape of the tumor to move and modulate the intensity of the beam to deliver varying doses of radiation to specific regions of the tumor. However, there are concerns that while the delivery of these high doses of radiation is more precise with IMRT, more healthy tissue may be exposed to low doses of radiation due to the higher number of angles irradiated [61]. Hall and Wu have estimated that the risk of SPMs following IMRT might be as much as double that of 3D-CRT [62].

Another change in the field of RT is the use of particle therapy (proton and carbon ion). The advantage of using protons in therapy is that most of the dose is delivered in a single area of the body, with limited exposure of the surrounding healthy tissue. In addition, the rationale is that some tumors would be resistant to low LET radiation from photons but susceptible to high LET radiation from protons and ions. Low LET radiation induces cell death mainly through indirect effects, such as the production of reactive oxygen species while high LET radiation causes irreparable DNA breaks that induce apoptosis. In a recent meta-analysis, Leroy and colleagues reviewed data from proton therapy from a total of 650 pediatric patients who had undergone proton therapy and found that the evidence was insufficient to conclude that proton therapy had any advantage in terms of effectiveness over photon therapy [63]. Another novel type of RT involves the use of carbon ions. Carbon ions are predicted to have a higher relative biological effectiveness than protons and thus greater tumor killing ability, but these claims have not yet been substantiated in clinical settings [60]. Proton and carbon ion therapies nonetheless do cause less acute toxicity than conventional treatment, and models calculate that they should cause fewer SPMs [64].

Alternative therapeutic options are also more commonly considered and the rates of RT usage have been steadily declining. The National Cancer Institute is the curator of the Surveillance, Epidemiology, and End Results (SEER) registry which collects information on cancer patients for the US [65]. Jairam and colleagues analyzed the data and found clear downward trends for the utilization of RT between 1973 and 2008 [66]. For example, there was an 80% decrease in the proportion of pediatric patients with acute lympholytic leukemia receiving RT, and a 74% decrease for NHL. There was also a decrease in RT use, although more modest, in most solid tumors [66]. On the other hand, the use of RT has remained high for HL, with about 72% of pediatric patients being treated with RT. Chemotherapy is associated with an increased risk of secondary leukemia in pediatric patients with HL that surpasses that associated with RT [67,68]. In addition, the use of RT in HL has achieved a recovery rate of 90%. However, major changes have been implemented in the way RT is administered. As is the case with other cancers, the field of irradiation in HL is increasingly focused with the use of involved-field RT and, more recently, involved-site RT, sparing adjacent healthy tissue [69].

## 4. Exposure from Diagnostic Modalities: Concerns Associated with Computed Tomography (CT) Exposures

Based on knowledge gained from studies of accidental and therapeutic exposure to radiation, there has been much concern regarding medical radiation utilized as a diagnostic modality, such as computed tomography (CT) and X-rays, because of the ever-growing number of patients who are routinely exposed. It has been reported that in the last 30 years, the average radiation doses Americans are exposed to have doubled [70,71]. Among all types of medical radiation, CT has been recognized as the largest contributor to this increase, since the natural background and X-ray sources of exposure have not changed [8,71]. Recent reports clearly demonstrate that a small but significant risk of CT-associated cancers exist [72,73].

### 4.1. Cancer Risks Associated with CT Exposures

CT imaging is a valuable diagnostic modality widely used in medicine. It has been demonstrated to significantly improve diagnosis and management strategies to advance overall patient care [74].

It is estimated that about 1 million CT scans are performed annually in children 5 years of age and younger in the US. According to several survey reports, 3% to 11% of patients who undergo CT diagnostic examinations in Western Europe and North America are under 15 years of age [75,76]. Additionally, the proportion of childhood CT examinations is rapidly increasing. For instance, a 63% increase in requests for pediatric CT scans was reported in the period from 1991–1994 [77]. A 92% increase in abdominal and pelvic CT examinations in children under the age of 15 was reported between 1996 and 1999 [7]. Overall, according to a recent retrospective cohort study that involved 355,008 children and included data from five large health care markets in Arizona, Texas, Florida, and Wisconsin, almost 8% of children underwent at least 1 CT scan, and 3.5% underwent 2 or more CT scans [78]. While it is generally accepted that the benefits of medical radiation outweigh the risks, there is, however, considerable concern since the doses of radiation received during the CT are higher than those from X-rays, and because of the considerable potential for long-term effects of pediatric irradiation.

In 2001, Brenner and colleagues estimated the risks of radiation-induced fatal cancer from pediatric CT examinations [7]. According to their calculations, CT scans (head and abdomen) during childhood resulted in a 10-fold increase in estimated risk compared to CT scans in adults, with estimated risks for abdominal CT examinations being significantly greater than those for head CT scans. In particular, the estimated life-time increase in cancer mortality risks associated with CT scans for 1-year old children are 0.18% and 0.07% for the abdomen and head, respectively [7]. A more recent study performed by Pearce and colleagues [79] was the first to report the risks of leukemia and brain tumors associated with pediatric CT examination. In this retrospective cohort study of almost 180,000 patients, the authors reported increased rates of leukemia and brain cancers after pediatric CT examinations with an average follow-up period of 10 years. Their study showed that cumulative absorbed organ doses of about 50 mGy delivered with CT to children tripled the risk of leukemia compared to doses below 0.5 mGy. Similarly, cumulative absorbed organ doses of 60 mGy associated with pediatric CT tripled the risk of brain cancer [79]. Based on the results of this epidemiological study, as well as leukemia and brain cancer incidences, it was estimated that 10 years after the first CT scan for patients younger than 10 years of age, 1 excess case of leukemia and 1 excess case of brain cancer per 10,000 head CT scans will occur [79]. Similar increases or projected risks in leukemia, brain, and other solid cancers were reported in the US [80], Taiwan [81], and Australia [82]. These cumulative absolute risks are relatively small. However, previous studies on exposed populations have shown that development of radiation-induced cancers may not occur until 20 to 40 years after exposure [38,83]. Therefore, two studies have estimated that about 30% of radiation-induced leukemias have not yet appeared within the extant 10-year period [38,83]. This number reaches as high as 90% in the case of brain cancers. Taking this into account, the estimated life-time risks of pediatric CT-associated leukemia might become 1-in-7500, and brain cancer 1-in-1000 [73].

However, at least one other study failed to identify any increase in cancer risk linked to CT scans in children [60]. In this work by Journy and colleagues on French children, the authors took into account the information available about the children prior to their first CT scan. This allowed the authors to exclude cases where cancer was present before the initial CT scan or who underwent CT scanning for a cause linked to a later cancer diagnosis. At least two recent studies have adopted this methodological approach and were still able to identify an increased risk associated with CT scans in childhood, albeit smaller than in the studies that did not [84,85]. One of these studies re-analyzed the data from Pearce and colleagues and found a more modest, yet still significant increase in cancer risk [85]; this raises doubts about possible additional biases in the experimental design of all epidemiological studies on the topic [86,87,88].

It must be emphasized that the first epidemiological study evaluated only two types of cancers, both of which have “shorter-than-typical latency periods in irradiated children” [73]. However, numerous reports discussed earlier in this review indicate that exposure to ionizing radiation in childhood, even at considerably low doses, may result in increased life-time risks of radiation-induced malignancies. These studies raise concerns regarding the possible effects of localized low-dose exposure in childhood on the breast and liver cancer rates in adulthood, especially after exposures received from chest and abdominal CTs.

### 4.2. Actions Taken

The knowledge obtained from these studies emphasizes the importance of judicious use of CT scans and other imaging procedures in children. The FDA issued its first set of recommendations 15 years ago as a Public Health Notification “Reducing radiation risk from computed tomography for pediatric and small adult patients”. In this document, several specific recommendations were given for the optimization of CT settings, including tube current modulation, reduction of the number of multiple scans, and elimination of inappropriate referrals for CT [89]. It was followed in 2010 by the White Paper “Initiative to reduce unnecessary radiation exposure from medical imaging”, where the particular issues associated with pediatric CT examinations were discussed further [90]. The As-Low-As-Reasonably-Achievable (ALARA) group devoted its 2001 conference to the risks of CT irradiation and dose reduction strategies for pediatric examinations [91]. Over the last decade, a number of professional organizations, such as the Society for Pediatric Radiology and the Radiological Society of North America, participated in numerous education forums, dedicated to pediatric applications of CT examinations. Additionally, the Alliance for Radiation Safety in Pediatric Imaging, a group tightly associated with the Image Gently and Image Wisely educational campaigns, was organized in 2007 [92,93]. This organization promotes radiation safety for children and consists of over 60 national and international professional societies and agencies. In particular, a cooperative initiative “to promote safe use of medical imaging devices, support informed clinical decision making and increase patient awareness” was announced. In addition to targeting health care providers, the FDA also released a consumer update destined for parents, entitled: “Radiology & Children: Extra Care Required” [94]. Further plans include the issuance of particular requirements for manufacturers and the introduction of quality assurance practices into the mandatory accreditation process of imaging facilities and hospitals [95]. Despite more than a decade of advocacy, it is still uncertain how much of an impact these initiatives have had on clinical practice. The number of CT scans performed on children in the US is still up from 2001, especially in the emergency rooms of the adult centers where most of the pediatric scans are administered [96,97,98]. A few studies have nevertheless reported a decrease in CT scan usage in pediatric patients overall in the UK and in hospitals for children in the US [99,100,101].

### 4.3. Future Considerations and Tasks

The first epidemiological studies provided valuable information regarding the potential effects of exposure to low dose radiation sources, such as CT. However, these studies are limited to short-term consequences, since the average follow-up period was only 10 years after CT examinations, allowing researchers to observe only the malignancies with the shortest latency periods. Clearly, further epidemiological studies that take into account the longer latency periods and other types of malignancies are needed to delineate the long-term consequences and life-time risks associated with CT examinations in childhood.

Another challenge is that the majority of CT scans in children are being performed in adult hospitals and centers. According to the results of the National Hospital Ambulatory Medical Care Survey performed between 1995 and 2007, about 90% of all pediatric emergency room CT examinations in the US were performed in adult hospitals [102]. Such examinations are often performed using adult settings, exposing the children to unnecessarily high levels of ionizing radiation. This requires implementation of new educational strategies and changes of practice in adult-focused hospitals where pediatric CT scans are performed [93].

There is also a need to determine specific indications for CT utilization in children. It has been proposed that CT examination should be considered in cases of acute brain trauma, suspected pulmonary interstitial and renal calculus diseases, and some skeletal pathology [9]. In all the other cases, utilization of non-ionizing image modalities should be given preference [6,9]. At the same time, the trend towards increased utilization of MRI and ultrasound in the last several years within pediatric institutions in the US has been reported [103,104].

Finally, there is an urgent need for the optimization of pediatric CT examinations for specific scan indications. The task is to “determine an effective organ dose range that balances image quality and image noise and then to manipulate tube current and peak kilovoltage while conforming to that range” [105]. This will likely be the most complicated task and will require cooperation between the pediatricians, pediatric radiologists, imaging physicists, and the industry at large.

## 5. Conclusions

Children are at a greater risk than adults to develop cancer after being exposed to radiation. Increases in the rates of leukemias and thyroid cancers associated with childhood exposure to radiation from A-bomb explosions, nuclear power plant explosions, and medical procedures have been well documented. Administration of potassium iodide in the hours following exposure to ^131^I is recommended to reduce the risk of thyroid cancer in children under the age of 15. RT methods in pediatric oncology are constantly evolving to maximize recovery while minimizing long-term cancer risks. The use of CT scans has been increasing in children and evidence is accumulating that indicates an association with an increase in cancer incidence. Guidelines have been developed recommending limiting the number of scans, using alternative imagery techniques, as well as tailoring radiation doses to the smaller size of children to provide care while lowering cancer risk.
